# Selection signature analysis using whole genome resequencing data reveals candidate genes for white plumage color in Korean native ducks

**DOI:** 10.5713/ab.24.0718

**Published:** 2025-02-27

**Authors:** Jaewon Kim, Jaegwon Kim, Eunjin Cho, Sunghyun Cho, Minjun Kim, Won-Hyung Chung, Jung-Woo Choi, Hyo Jun Choo, Jun Heon Lee

**Affiliations:** 1 Division of Animal and Dairy Science, Chungnam National University, Daejeon, Korea; 2 Department of Animal Science, College of Animal Life Sciences, Kangwon National University, Chuncheon, Korea; 3 Department of Bio-AI Convergence, Chungnam National University, Daejeon, Korea; 4 Research and Development Center, Insilicogen Inc., Yongin, Korea; 5 Poultry Research Institute, National Institute of Animal Science, Rural Development Administration, Pyeongchang, Korea

**Keywords:** Korean Native Duck, Plumage Color, Population Analysis, Selection Signature Analysis, Transposable Element, Whole Genome Resequencing

## Abstract

**Objective:**

Domestication alters the phenotypes of wild animals to meet human demands and leaves characteristic patterns in their genomes. Various selection signature analysis methods have been developed to identify these characteristic patterns left in the genome. The Korean native duck (KND) is one of the domesticated species in Korea. KND is categorized into two populations based on plumage color; colored KND (KNDC) and white KND (KNDW). To enhance the competitiveness of native ducks, it is necessary to establish a KNDW line. In this study, we conducted selection signature analysis to identify candidate genes associated with white plumage color in KNDs.

**Methods:**

We generated whole genome resequencing data from 22 KNDCs, 22KNDWs, and 10 Pekin ducks (PKDs). To detect distinct genomic regions between KND populations with different plumage colors, we analyzed three types of selection signature analysis: the fixation index (Fst), nucleotide diversity(π), and cross-population extended haplotype heterozygosity (XP-EHH).

**Results:**

Population structure analysis showed that although KNDC and KNDW are distinct from PKD, they form a single group sharing a common ancestor. The results of Fst and π analyses revealed that compared to KNDC, there were strong selection signals in the *MITF* gene in KNDW, with a 6,641 bp insertion in the intron 2 region. This variant is a transposable element insertion that causes white plumage in PKD. In addition, XP-EHH analysis identified *DCT*, *KIT*, *TYR*, and *ADCY9* as major candidate genes associated with pigmentation in the KND population.

**Conclusion:**

White plumage in KNDW is caused by a transposable element insertion in the *MITF* gene. This finding improves our understanding of plumage color in KND and supports the establishment of KNDW breeding programs.

## INTRODUCTION

The domestication process of animals for humans has a long history, with origins tracing back thousands of years. Through this process, ancestral animals underwent strong artificial selection for economic traits that were desirable for humans. These rigorous and deliberate selection events created remarkable features in the genomes of domesticated livestock, distinguishing them from those found in wild animals [1]. Genomic regions associated with artificially selected phenotypes exhibit unique characteristics, such as strong linkage disequilibrium, reduced genetic diversity, increased frequency of specific alleles, long haplotypes, and higher homozygosity [2]. These characteristics may be applied to identify genomic regions associated with specific phenotypes, in selection signature analysis [3]. Recent advancements in genomics, including high-throughput DNA sequencing, high-density single-nucleotide polymorphism (SNP) chips, and advanced statistical tools, have led to the development of various methodologies for identifying genomic regions. The fixation index (Fst) measures the differences in allele fixation rates between populations, rather than within a population [4,5]. The Fst is usually determined for sliding windows to examine continuous SNPs, because positive selection tends to create linkages with neighboring alleles [6]. Nucleotide diversity (π) is calculated based on comparing the average numbers of nucleotide differences between DNA sequences sampled from the same population [7]. Regions with lower genetic diversity can be identified by comparing the π values across different populations [8,9]. Another approach for detecting selection signatures is to analyze extended haplotype homozygosity (EHH) created by linkage disequilibrium. EHH is computed as the probability of homozygosity for a core haplotype that contains a beneficial allele [10]. Cross-population EHH (XP-EHH) can identify regions under selection specific to certain populations [11]. These three selection signature analysis methods, based on diverse approaches and statistical techniques, can be used to detect different selective sweeps, in which variation in linked neutral sites is reduced by a new advantageous mutation when it increases in frequency within the population [3]. To perform comprehensive selection signature analysis, it is important to apply more than one of these methods and compare the results.

Ducks are among the most economically important species, as their meat, eggs, and feathers contribute significantly to the livestock industry [12]. Domesticated ducks are known to have originated from mallards in central China, with a history that began approximately 2,500 years ago, which is relatively recent compared to other domesticated animals. Nevertheless, the intensive artificial selection process has led to various phenotypic changes that are markedly distinct from those of mallards [8,13]. For example, the Pekin duck (PKD), exhibits rapid growth and white plumage that is used as down in clothing and quilts, which makes it the predominant duck breed used in the global duck industry [14]. Previous studies based on whole genome-wide analysis have discovered several genes that contribute to plumage color (e.g., *MITF*) [14,15], and body size (e.g., *IGF2BP1*, *NR2F2*, *DCN*, *SGCZ*, *SGCG*, and *IGF2R*) [14–17], which have played key roles in significantly differentiating domesticated ducks from mallards.

The Korean native duck (KND) is a domesticated breed that has adapted to the Korean climate [18]. The KND is thought to have originated from a crossbreeding between migratory mallard ducks and indigenous PKDs, developed by the National Institute of Animal Science (NIAS) in 1997; however, there are no precise records documenting its development [19]. KND breeds are categorized into two types based on plumage color: the colored KND (KNDC), which has a black-brown color, and the white KND (KNDW). The KNDW was selected from white individuals that emerged from the fertilized KNDC eggs [20]. Although the KND has high beneficial fatty acid content and unique meat flavor, it constitutes only 10% of the domestic duck industry [21]. This low percentage is due to their slower growth rates and small body size compared to commercial ducks [18]. The KNDC is predominant among KNDs, and its colored feathers are more difficult to remove during the feather plucking process compared to white feathers, posing a significant challenge for the commercial poultry meat production industry [20,22]. To address this issue, NIAS initiated a project in 2014 to develop a white native duck line aimed at improving uniformity, meat quality, and genetic characteristics. However, the project was halted in 2015 due to an outbreak of highly pathogenic avian influenza [23]. Notwithstanding these difficulties, the potential benefits of re-establishing and refining KNDW breeding strategies remain significant for enhancing their competitiveness in the poultry industry. However, the genomic characterization of the KND remains insufficient, underscoring the need for further research [24]. In this study, we used high-depth whole-genome resequencing data (30X) from 44 KNDs (22 KNDCs and 22 KNDWs) and 10 PKDs to conduct comprehensive selection signature analysis using various methods (Fst, π, and XP-EHH). We identified the causal gene responsible for determining the white plumage color in KND populations through comparative analysis between KNDCs and KNDWs.

## MATERIALS AND METHODS

### Ethical approval

The experimental procedures were approved by the Institution of Animal Care and Use Committee of the National Institute of Animal Science (NIAS-203-617).

### Sampling and DNA extraction

Blood samples were collected from 22 KNDCs, 22 KNDWs, and 10 PKDs, which were provided by the NIAS in Korea. All blood samples were obtained from the brachial vein. We used the PrimePrep Genomic DNA Extraction Kit from Blood (GeNetBio, Daejeon, Korea) for genomic DNA (gDNA) extraction and verified the gDNA using a NanoDrop 2000c spectrophotometer (Thermo Fisher Scientific, Waltham, MA, USA). The gDNA was stored at −20°C until resequencing.

### Library preparation and resequencing

Paired-end gDNA libraries were generated by using TruSeq Nano DNA next-generation sequencing library preparation workflow (Illumina, San Diego, CA, USA), and the generated libraries were sequenced on an Illumina NovaSeq 6000 platform (Illumina) to obtain 151 bp paired-end reads.

### Variant calling and quality control

Variant calling was performed by aligning the reads to the reference genome ZJU1.0 (accession no. GCF_015476345.1) using BWA v0.7.17 [25]. The aligned BAM files were sorted using SAMtools v1.19 [26]. To eliminate polymerase chain reaction (PCR) duplicates, we employed the MarkDuplicates tool in GATK v4.4.0. Variant calling was subsequently conducted using the HaplotypeCaller tool in GATK, generating individual gVCF files, which were subjected to joint genotyping to produce a consolidated VCF file. Variants were filtered using the VariantFiltration tool in GATK, with the following criteria: QD<3.0, FS>30.0, MQ<30.0, DP<7, GQ<10.0, MQRankSum<−2.0, and ReadPosRankSum<−2.0. To isolate only biallelic SNP variants, we applied VCFtools v0.1.15 [27], resulting in the filtering of 22,720,731 SNPs. For more accurate analysis, we separated the SNPs by population and conducted separate quality control using PLINK v1.9 [28], based on the following criteria: SNP call rate<90% (--geno 0.1), sample call rate<90% (--mind 0.1), and Hardy–Weinberg equilibrium<10^−6^ (--hwe 1e-6). Four KNDC samples were removed during this step due to their low sample call rate. Subsequently, the filtered SNPs were combined to extract only those SNPs were common across the entire population. A total of 20,274,385 SNPs were retained for downstream analysis.

### Population structure analysis

To assess the genetic relationships of each population, we conducted principal component analysis (PCA) and admixture analysis. SNPs used in the population analysis underwent an additional quality control filtering step, in which SNPs with a minor allele frequency < 0.01 were filtered out from each population, and common SNPs across the three populations were extracted using PLINKv1.9 [28]. In total, 9,398,767 common SNPs were obtained for subsequent analyses. The PCA was conducted using PLINK v.1.9, and all three principal components were used to distinguish the population structure [28]. Admixture analysis was conducted using the ADMIXTURE software, with the number of random common ancestral clusters (K) values ranging from 2 to 5 [29].

### Selection signature analysis

We conducted comprehensive pairwise selection signature analysis by performing Fst, π, and XP-EHH analyses. The top 1% of values of the entire distribution window were defined as the threshold for significant selection regions. VCFtools v0.1.15 was used to calculate Weir & Cockerham’s weighted Fst and π values [5,27]. The sliding window and step sizes were set to 20 and 10 kb. The Fst values were transformed into Z scores as follows: *Z_Fst_* = ((*Fst* − *μFst*)/*σFst*) [18]. The log2 π ratio was calculated as follows: log2 π ratio = log2 (π [B]/π [A]), using in-house scripts. A positive log2 π ratio indicates a region of reduced genomic diversity in population A, while a negative value indicates reduced genomic diversity in population B. Windows exceeding the top 1% of results were designated as significantly selected regions. XP-EHH analysis was performed using rehh v3.2.2 package in R [30]. To improve the statistical power of selection detection, we reconstructed haplotypes using Eagle software v.2.4.1, except for imputing missing genotypes [31]. When calculating EHH score for XP-EHH, information on polarized alleles, such as their ancestral or derived states, is required. However, as this information was unavailable for our dataset, XP-EHH was conducted using the “unpolarized” option, such that computations were based on major and minor alleles [32]. After calculating the XP-EHH values, we scanned the genomes by using a 20 kb sliding window size and a 10 kb step size to detect significantly selected regions, which were defined as those where the average normalized XP-EHH scores in each window exceeded the top 1% of values. Genes within all significantly selected regions were annotated based on the ZJU1.0 gtf file.

### Detection of structural variants in *MITF*

Through the selection signature analysis, we found highly differentiated patterns in the *MITF* gene regions. To investigate structural variants (SVs) in *MITF*, we extracted the chromosome 13 mapping file (BAM) using Samtools v1.7 [33] and visualized it using the Integrated Genome Viewer (IGV). SV genotypes were detected using Delly software v0.8.7 [34,35].

## RESULTS

### Genome resequencing and variant calling

We extracted gDNA from 54 ducks, including 22 KNDWs, 22 KNDCs, and 10 PKDs, provided by the NIAS. We generated approximately 16.774 billion paired-end reads with a read length of 151 bp. This process resulted in a total of 1.2 tb of sequencing data, providing an average coverage of 96.42% across the duck reference genome (ZJU1.0). The average sequencing depth was 32.06-fold, ensuring sufficient resolution for reliable variant detection. The ZJU1.0 reference genome for ducks is indeed widely used; however, its unresolved contigs limit our ability to fully understand the genome structure due to the incomplete representation of genomic features. Therefore, we focused on the autosomal chromosomes in the downstream process to gain more accurate insights into the genetic landscape of the Korean duck population. We also conducted duplication removal to minimize potential bias caused by PCR duplicates. The average duplication rate across samples was calculated to be 9.44%. After removing duplicates, the remaining reads provided a high mapping rate (≥99%) and a minimum sequencing depth of 30-fold, which was sufficient for high-confidence variant calling. Variant calling was performed for each sample using the HaplotypeCaller tool in GATK in GVCF mode, producing individual gVCF files, which were subjected to joint genotyping, resulting in a consolidated raw VCF file containing 33,275,264 variants across all samples. To ensure accurate analysis, we conducted additional population-specific variant filtering, resulting in 20,274,385 high-quality common SNP data from the three populations.

### Population structure in Korean native duck

**Figure 1:**
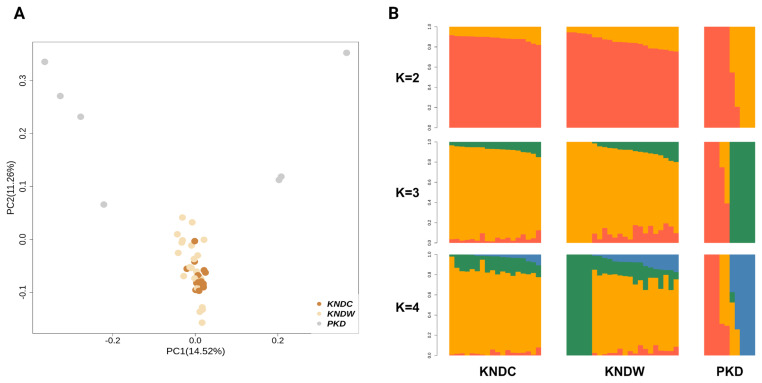
Population genetic structure analysis. (A) Principal component analysis (PCA) result plot for the first and second components. (B) Admixture analysis results of KNDC, KNDW, and PKD with K = 2 to K = 4. KNDC, colored Korean native duck; KNDW, white Korean native duck; PKD, Pekin duck.

PCA and admixture analysis were performed to determine genetic differences among the KNDC, KNDW, and PKD populations (Figure 1; Supplement 1). The PCA results showed that KNDCs and KNDWs were completely clustered together, and clear separation from PKDs. However, PKDs did not form a distinct cluster, but instead divided into two clusters based on principal components. These results were supported by the admixture results, which revealed that KNDs (KNDCs and KNDWs) were clearly separated from PKDs, and that PKDs were divided by two groups (K = 2). At K = 3, which had the lowest value of cross-validation error, KNDCs and KNDWs still showed similar ancestral composition. This result indicates that these two populations share a common ancestry. As the K value increased, KNDW individuals were segregated into two clusters, whereas PKD split into three clusters. These ancestry patterns may reflect lineage differences within the three populations.

### Selection signatures based on allele differentiation

**Figure 2:**
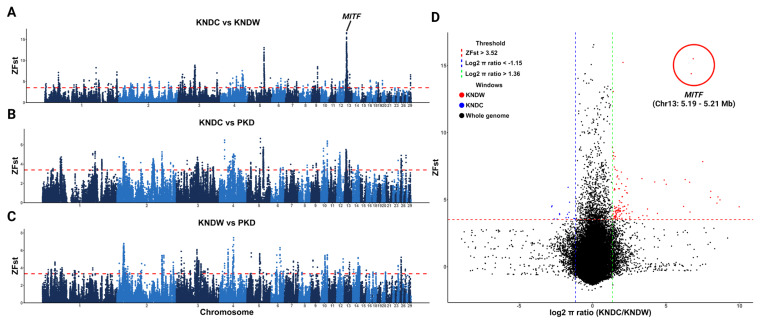
Selection signature analysis based on differentiated allele frequency in duck populations. Z-transformed Fst (Z_Fst_) and nucleotide diversity (π) ratio were scanned using a 20 kb sliding window with a 10 kb step size. The dashed lines represent the top 1% values of Z_Fst_ and π ratio. (A) Z_Fst_ results between KNDC and KNDW (Z_Fst_>3.52). (B) Z_Fst_ results between KNDC and PKD (Z_Fst_>3.39). (C) Z_Fst_ results between KNDW and PKD (Z_Fst_>3.34). (D) Z_Fst_ results with π ratio between KNDC and KNDW (Z_Fst_>3.52, log2 π KNDC/KNDW>1.36, and log2 π KNDC/KNDW<−1.15). KNDC, colored Korean native duck; KNDW, white Korean native duck; PKD, Pekin duck. Figure created with permission from BioRender (https://biorender.com/).

**Figure 3:**
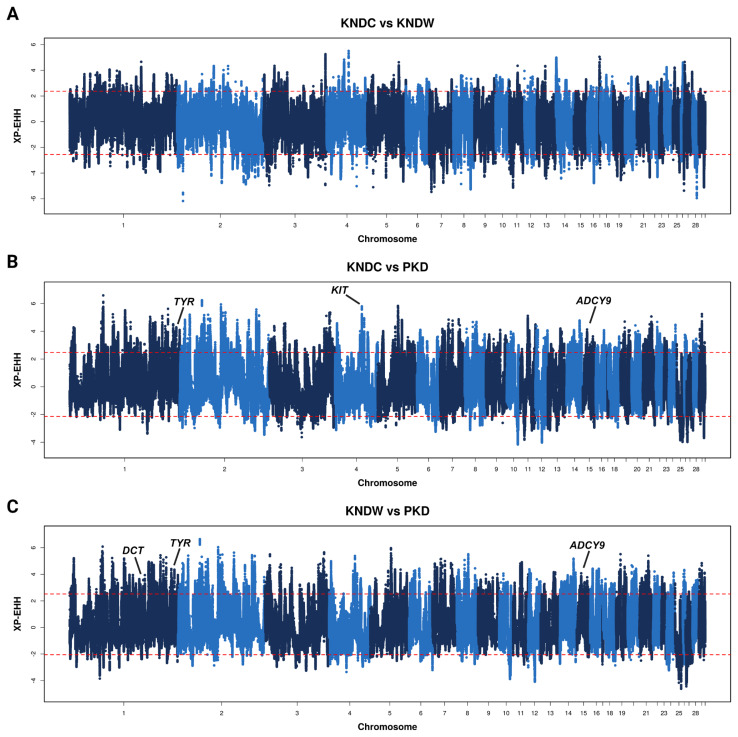
Selection signature analysis based on extended haplotype in duck populations. XP-EHH was scanned using a 20 kb sliding window with a 10 kb step size. The dashed lines represent the top 1% values of XP-EHH. (A) XP-EHH results between KNDC (XP-EHH>2.37) and KNDW (XP-EHH<-2.55). (B) XP-EHH results between KNDC (XP-EHH>2.47) and PKD (XP-EHH<−2.13). (C) XP-EHH results between KNDW (XP-EHH>2.52) and PKD (XP-EHH<−2.05). KNDC, colored Korean native duck; KNDW, white Korean native duck; PKD, Pekin duck. Figure created with permission from BioRender (https://biorender.com/).

To identify regions where allele frequencies have been differentiated by strong selection, we compared the genomes of KNDWs and KNDCs using Z-transformed Fst (Z_Fst_) and π methods with a 20 kb window size and 10 kb step size. In the Z_Fst_ analysis comparing KNDCs with KNDWs, we identified 335 genes (Z_Fst_>3.52) in the top 1% of Z_Fst_ values; the most significantly differentiated regions were located on chromosome 13 (Figure 2A). Comparing PKDs with KNDCs and KNDWs, we identified 264 (Z_Fst_>3.39) and 293 genes (Z_Fst_>3.34) in the top 1% of Z_Fst_ values, respectively (Figures 2B, 2C). Although Fst analysis identifies regions where genetic diversity differs between two populations, it cannot specify which regions have been subject to selection in a particular population. To identify regions where genetic diversity has decreased in each population due to positive selection, we conducted both Z_Fst_ and π methods. In the significant genetic regions exceeding the top 1% of Z_Fst_ and π ratio values between KNDCs and KNDWs, we identified three genes (Z_Fst_>3.52 and log2 π _KNDC/KNDW_>1.36) in KNDCs and 44 genes (Z_Fst_>3.52 and log2 π _KNDC/KNDW_<−1.15) in KNDWs, respectively (Figure 2D). Among these genes, microphthalmia-associated transcription factor (*MITF*) gene regions (chromosome 13: 5.19 to 5.22 Mb) were significantly differentiated in KNDWs. In the comparison between PKD and KND populations, the *MITF* gene did not appear in significant selection regions when compared to KNDW, but was identified in PKDs compared to KNDCs (Supplement 2). Furthermore, we performed the XP-EHH method, based on extended haplotype, with a 20 kb window size and a 10 kb step size to detect selection regions associated with plumage color. The results of a comparison between KNDCs and KNDWs revealed that 210 genes (XP-EHH>2.37) were positively selected in KNDCs, and 236 genes (XP-EHH<−2.55) were selected in KNDWs; however, there were no genes significantly related to pigmentation (Figure 3). Compared to PKDs, we identified 175 genes (XP-EHH>2.47) in KNDCs and 171 genes (XP-EHH>2.52) in KNDWs (Figure 3). Interestingly, among the significant genes of the KND populations, several genes were related to pigmentation in KNDCs (*TYR*, *KIT*, and *ADCY9*) and KNDWs (*DCT*, *TYR*, and *ADCY9*), with *TYR* and *ADCY9* were identified in both populations.

### Structural variants in *MITF* between colored and white ducks

**Figure 4:**
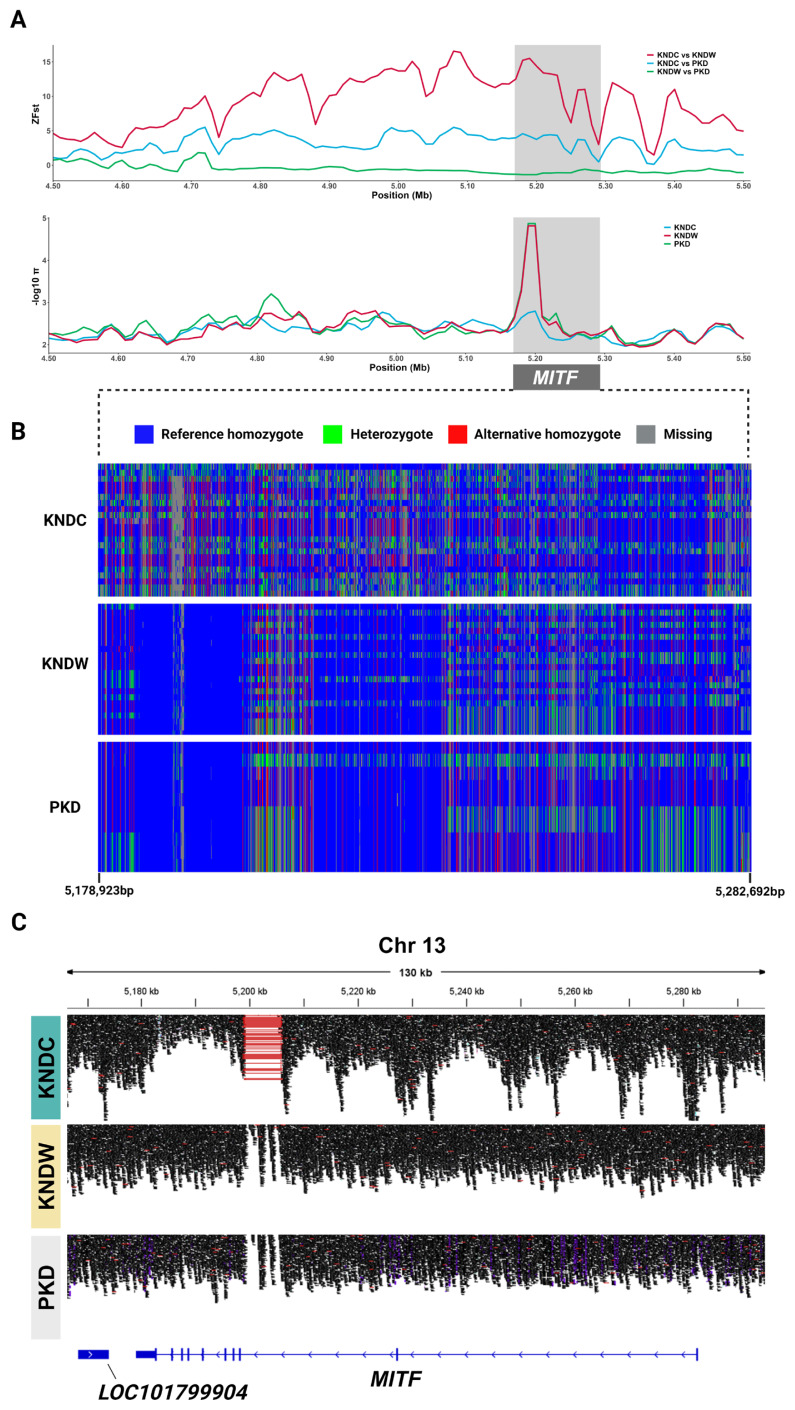
Identification of significant selection signal in *MITF* gene (chromosome 13: 5,178,923–5,282,692 bp). (A) Plot of Z-transformed Fst (Z_Fst_) and nucleotide diversity (π) values around MITF gene region (chromosome 13: 4.5–5.5 Mb). (B) Haplotype comparison analysis of *MITF* gene across the duck populations. (C) Integrate genomic viewer (IGV) visualization of *MITF* gene for 6.6 kb deletion in KNDCs. The 6.6 kb deletion was located in the intron 2 region of *MITF* in KNDCs. KNDC, colored Korean native duck; KNDW, white Korean native duck; PKD, Pekin duck. Figure created with permission from BioRender (https://biorender.com/).

Our Z_Fst_ and π analyses revealed that the *MITF* gene was highly differentiated between KNDCs and KNDWs. Notably, the *MITF* gene region exhibited more significant selection in white ducks than in colored ducks (Figure 4A). To verify the significant allele frequencies in the *MITF* gene region, we performed a haplotype comparison analysis of these populations. Interestingly, KNDCs exhibited significantly higher genetic diversity than the other populations, whereas KNDWs and PKDs showed relatively fixed genetic diversity and similar haplotype patterns (Figure 4B). Moreover, the most significant window regions (chromosome 13: 5.19 to 5.22 Mb) in the Z_Fst_ and π analyses comparing KNDCs and KNDWs contained extensive missing alleles among KNDC haplotypes. Read mapping data from the *MITF* region of the three populations were visualized using IGV, and we observed that a long intronic deletion in intron 2 appeared only in the KNDC (Figure 4C). Using the SV detection tool Delly, we confirmed that the length of the deletion SV was 6,641 bp (NC_051784.1:g.5199182_5205823del); this deletion was completely absent in the white plumage populations (KNDW and PKD) (Table 1). Previous studies have reported that this SV is an insertion that causes white plumage color in PKDs [8,14,15]. A recent study also revealed that this insertion is a transposable element (TE) belonging to the Gypsy superfamily [15]; the 3′ long terminal repeat region of this TE contains a promoter element that interacts with transcriptional factors. When the TE was inserted into the *MITF* gene, it produced a new *MITF* transcript instead of the MITF-M transcript, which is involved in melanin production in ducks [15]. However, our results identified it as a deletion because the reference genome is based on the PKD, such that the absence of the TE insertion in KNDCs appears as a deletion when compared to the PKD reference genome. Thus, our results suggest that white plumage color in KNDWs is caused by a TE insertion in the intron 2 region of *MITF* that leads to white plumage color in PKDs.

## DISCUSSION

Plumage color is considered a valuable economic trait in the poultry industry, as it distinguishes each breed and influences consumer preferences [36]. Although several genes involved in plumage color in ducks have been identified, the gene responsible for the white plumage in native ducks has not yet been discovered. Therefore, we conducted comparative selection signature analysis on three duck populations (KNDCs, KNDWs, and PKDs) to identify candidate genes associated with white plumage color in KNDs. First, we conducted a population structure analysis to determine whether KNDCs and KNDWs share a common ancestry. Both PCA and admixture analysis results showed that KNDC and KNDW cluster together, and their shared genomic pattern suggested that they represent a single group that differs only in plumage color. The admixture results demonstrated a more heterogeneous pattern in KNDs compared to PKDs, likely due to the historical background of crossbreeding between mallard ducks and PKDs in the KND population. Unlike KNDCs, some KNDW individuals exhibited an independent ancestry pattern starting from K = 3, which suggests that KNDWs may have been selected as a distinct line with a unique genetic composition within the KND population. Allele frequency-based analyses (Fst and π) showed that the *MITF* gene region exhibited high allele diversity in KNDCs, but fixed allele frequencies in white duck breeds, which is consistent with a previous report of significant differentiation in this region between KNDs and PDs [24]. *MITF* is a transcription factor that regulates the expression of various genes involved in melanin synthesis in melanocytes. The *MITF* gene can be transcribed into various isoforms, among which the MITF-M isoform is specifically expressed in melanocytes and plays a crucial role in the melanin synthesis [37]. Previous studies have reported that variation affecting the expression of MITF-M is associated with plumage color in ducks [38–40]. Interestingly, we found that the *MITF* gene region in KNDWs contains a TE insertion previously identified to contribute to white plumage in PKDs [15]. When the TE insertion mutation was heterozygous, the ducks exhibited colored plumage, whereas only homozygous individuals displayed white plumage, indicating that this variant behaves in a recessive manner [15]. Changes in plumage color due to TE insertions are commonly observed in various animal species. TEs can be inserted into various other genes such as *ASIP*, *EDNRB*, *TYR*, and *LYST*, leading to the creation of stop codons, new transcripts, splicing alterations, or domain function modifications, all of which can disrupt the melanin synthesis pathway [41]. Based on these findings, we speculate that the TE-inserted *MITF* allele was introgressed into the KND population via hybridization between mallards and PKDs. Through the XP-EHH analysis, we identified genes in the KND populations that are involved in melanogenesis, including *DCT*, *KIT*, *TYR*, and *ADCY9*. *DCT* is a gene encoding a tyrosinase-related protein, and variation within this gene is known to be associated with plumage color in ducks [42]. *KIT* is a gene that encodes a tyrosine kinase receptor and interacts with a stem cell factor to regulate melanogenesis [43]. A deletion mutation in the *KIT* gene has been shown to play a role in determining plumage color in geese [44]. *TYR* encodes tyrosinase, a key enzyme in the melanin synthesis process, and is linked to beak color in ducks [45]. *ADCY9* is a gene involved in the conversion of adenosine 3′,5′-cyclic monophosphate (cAMP); cAMP signaling plays a crucial role in the melanin synthesis pathway [46]. The genes identified through XP-EHH analysis differed from those detected in the Fst and nucleotide diversity analyses, because these methods have different theoretical bases. XP-EHH analysis is more suitable for detecting recent selection events, whereas allele frequency-based indexes such as Fst and π are more effective for identifying signals of long-established or nearly fixed selection [47]. These results highlight the importance of conducting comprehensive selection signature analysis using multiple methods with different approaches, rather than relying on a single method, to obtain more reliable and robust results. It is likely that our XP-EHH analysis detected more color-related genes in both KNDCs and KNDWs than in PKDs because KNDs were artificially selected to preserve their distinct coloration characteristics.

In summary, we performed a comprehensive analysis to identify the genetic basis of white plumage color in KNDs. Our findings suggest that the white plumage color in KNDWs is caused by a TE insertion in the *MITF* gene, which is the same causative mutation found in PKDs. This finding supports the hypothesis that KNDs may have undergone hybridization with PKDs in the past. Additionally, our XP-EHH analysis highlighted other melanogenesis-related genes including *DCT*, *KIT*, *TYR*, and *ADCY9*, suggesting a complex genetic basis for plumage color. These findings reveal the genetic mechanisms of plumage color in the KND and support breeding strategies to identify heterozygous carriers for establishing and maintaining the KNDW line.

## Figures and Tables

**Table 1:** Genotype distribution of 6,641 bp deletion allele in the MITF intron 2 region

Population	Number of samples	Plumage color	Genotype		

			A/A	A/DEL^1)^	DEL/DEL
Colored Korean native duck	18	Color	-	2	16
White Korean native duck	22	White	22	-	-
Pekin duck	10	White	10	-	-

1) NC_051784.1:g.5199182_5205823del.

## SUPPLEMENTARY MATERIAL

**Supplement 1.** Population genetic structure analysis. **Supplement 2.** Selection signature analysis based on differentiated allele frequency in duck populations.
