# Supplementary material for: Selection signature analysis using whole genome resequencing data reveals candidate genes for white plumage color in Korean native ducks
Source: Anim Biosci. 2025 Feb 27;38(8):1594–604. doi: 10.5713/ab.24.0718 (PMC12229940; doi:10.5713/ab.24.0718)
Supplement: Supplementary file 1 [file ab-24-0718-Supplementary.pdf]

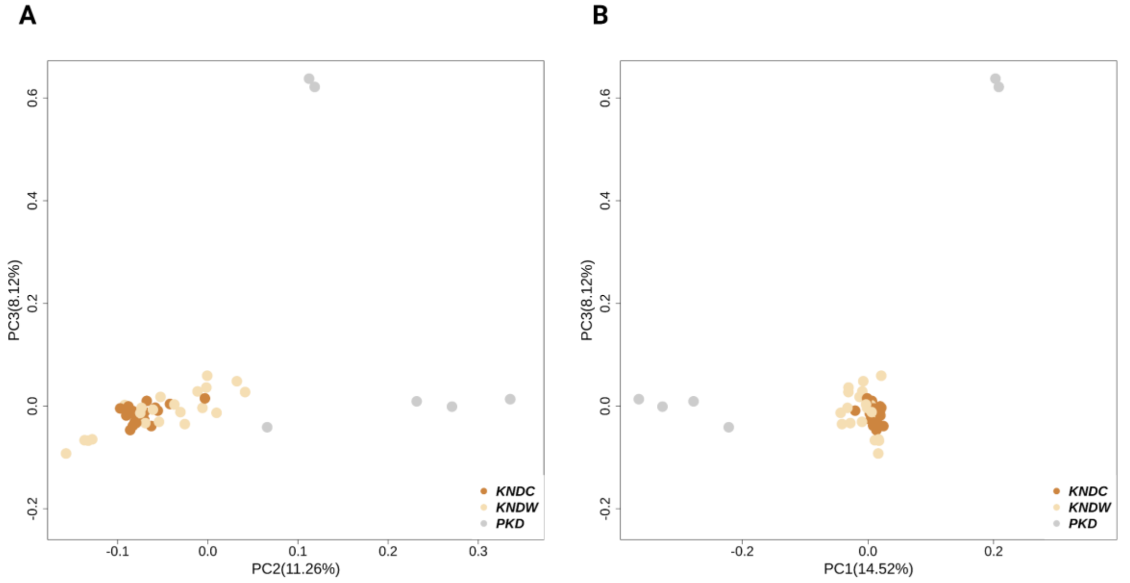

Supplement 1. Population genetic structure analysis. (A) Principal component analysis (PCA) result plot for the second and third components. (B) PCA result plot for the first and third components. Figure created with permission from BioRender (https://biorender.com/).

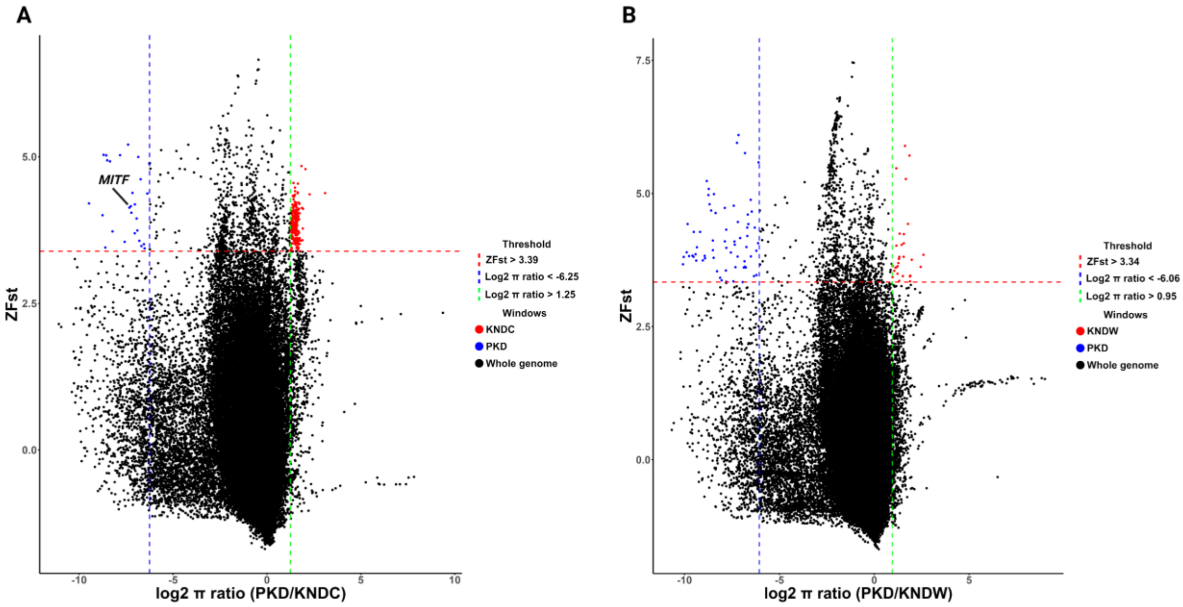

Supplement 2. Selection signature analysis based on differentiated allele frequency in duck populations. Z-transformed Fst (ZFst) and nucleotide diversity (π)-ratio were scanned using 20 kb sliding window with 10 kb step size. The dashed lines represent the top 1% values of ZFst and π-ratio. (A) ZFst results with π-ratio between KNDC and PKD (ZFst > 3.39, $\log_2 \pi_{PKD/KNDC} > 1.25$, $\log_2 \pi_{PKD/KNDC} < -6.25$). (B) ZFst results with π-ratio between KNDW and PKD (ZFst > 3.34, $\log_2 \pi_{PKD/KNDW} > 0.95$, $\log_2 \pi_{PKD/KNDW} < -6.06$). KNDC, colored Korean native duck; KNDW, white Korean native duck; PKD, Pekin duck. Figure created with permission from BioRender (https://biorender.com/).
